# The control rate of hypertension across months of year and hours of day in a large real-world database

**DOI:** 10.1038/s41440-024-01817-1

**Published:** 2024-08-21

**Authors:** Qinghua Yan, Minna Cheng, Wenli Xu, Yibang Cheng, Fei Wu, Yuheng Wang, Qinping Yang, Yan Shi, Jiguang Wang

**Affiliations:** 1 Division of Chronic Non-Communicable Disease and Injury, Shanghai Municipal Center for Disease Control and Prevention, Shanghai, China; 2 Department of Hypertension, Centre for Epidemiological Studies and Clinical Trials, the Shanghai Institute of Hypertension, Shanghai Key Laboratory of Hypertension, Ruijin Hospital, Shanghai Jiaotong University School of Medicine, Shanghai, China

**Keywords:** Blood Pressure, Hypertension, Hypertension Control, Office Blood Pressure Measurement

## Abstract

We investigated the control rate of hypertension across months of year and hours of day in a real-world database. The study participants were hypertensive patients from 142 community health centers across 16 districts in Shanghai, China, who measured their blood pressure with an automatic office blood pressure measurement platform between 2018 and 2023. The 343,400 hypertensive patients included 53.7% of women, and had average age of 70.2 (±8.1) years (range 50–90 years). For months of year, the control rate of hypertension was lowest in February and highest in August (51.9% vs 71.8%). For hours of day, the control rate of hypertension was lowest at 7:00 AM and highest at 12:00 PM (52.1% vs 76.0%). When the months of year and hour of day were considered together, the control rate was lowest at 7 AM in February (42.1%), and highest at 12 PM in July (86.8%). In 8516 patients who had uncontrolled blood pressure in the early morning and had their blood pressure also measured around noon, 45.7% had masked uncontrolled morning hypertension, with higher rates in spring and summer, and in women, those aged 50-69 years, and non-diabetic patients. The control rate of hypertension varies greatly across months of year and hours of day, suggesting that the evaluation of blood pressure control has to take into full consideration the measurement time in terms of months and hours.

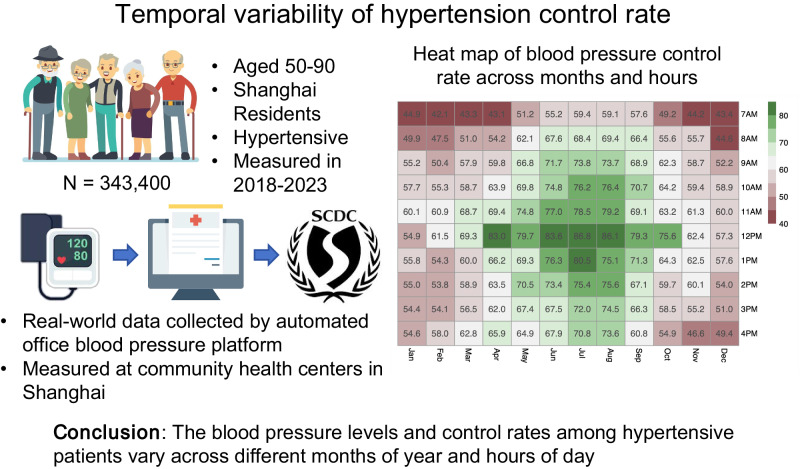

## Introduction

Hypertension is the leading cause of cardiovascular events, such as stroke, myocardial infarction, congestive heart failure, end-stage renal disease, and so on [1–5]. However, antihypertensive drug treatment may effectively prevent these cardiovascular events by lowering blood pressure, especially by controlling blood pressure to a target level, for instance, a systolic/diastolic of 140/90 mmHg and below [6]. That is why the management of hypertension is evaluated not only for treatment rate, but also control rate [7].

Current hypertension guidelines recommend the use of office blood pressure for the diagnosis and therapeutic monitoring [7, 8], although out-of-office blood pressure measurements, either ambulatory or home blood pressure monitoring, plays an increasing role in these regards [9, 10]. In fact, office blood pressure remains a major assessment for blood pressure control, especially with the increasing use of automated office blood pressure measurement platforms [11]. With this platform, office blood pressure measurement can be standardized in many aspects, such as number of measurements, resting time, time interval between consecutive measurements, and so on. However, office blood pressure can still vary across the measurement hours of day and months of year [12] for various reasons, such as ambient temperature [13, 14] and others [15]. There is indeed increasing awareness of the time of blood pressure measurement. Experts suggest that we may focus on blood pressure measurement in the morning for the evaluation of blood pressure control [16] and that we may have to watch for the seasonal variation in blood pressure [17].

In the present study, we analyzed a large sample real-world database to investigate blood pressure control according to office blood pressure measured in different months of year and different hours of day.

Point of view

- Clinical relevance
  The evaluation of blood pressure control has to take into hourly and monthly variation account in the management of hypertension.
- Future direction
  The results need to be confirmed in other populations, preferably with a focus on the relationship between the hourly and monthly variation and clinical outcomes.
- Consideration for the Asian population
  There are substantial differences in the degree of morning blood pressure surge between Asian and Western population, which calls for more attention and action on blood pressure variation in Asians.

## Methods

### Hypertensive patients

The study participants were hypertensive patients who participated in the National Basic Public Health Service - Management of Hypertension in Community program from June 2018 to September 2023 in Shanghai, China. For inclusion, they had to be 50 to 90 years of age and measured their blood pressure with an automated office blood pressure platform. Until the end of September 2023, 343,400 patients from 142 community health centers across all 16 districts of the city of Shanghai participated in the program, and a total of 1,654,072 measurements were obtained. For the primary analysis, we selected per participant blood pressure measurement on a single occasion. To enrich the number of patients who had their blood pressure ever measured between 7AM and 8AM, we selected the first blood pressure measurement in this time period. Otherwise, we selected the first ever blood pressure measurement in the rest hours. For a secondary analysis in a subgroup of patients with uncontrolled blood pressure between 7AM and 8AM, we further selected the nearest blood pressure measurement between 9AM and 4PM within the same season.

### Office blood pressure measurement

Blood pressure was measured with an automated office blood pressure platform, developed by the Shanghai Municipal Center for Disease Control and Prevention in collaboration with the Shanghai Institute of Hypertension at Ruijin Hospital, Shanghai Jiaotong University School of Medicine, Shanghai, China. At each participating community health center, the platform was established in a dedicated area with a comfortable indoor temperature. Validated automatic electronic blood pressure monitors were placed on tables with chairs properly adjusted to body height and with multiple-sized arm cuffs. Whenever a patient needed any help or encountered any problem, a trained volunteer was present to provide help for proper blood pressure measurement, including but not restricting to the choice of properly sized cuffs, adjustment of the height of chairs, and initiation of the measuring system.

Blood pressure was measured with a standardized procedure, after having rested for 5–10 min in a seated position. Blood pressure was measured twice consecutively with a one-minute interval. If the difference between the first two blood pressure readings, calculated automatically by the software, was ≥5 mmHg, a third blood pressure measurement was obtained. These two or three readings were automatically transmitted to a digital platform and averaged for clinical decisions and for the present analysis.

A quality control program was applied for the hardware and software system, measurement procedures, and training of the medical staff for the management of the system. Briefly, an expert panel evaluated the appropriateness of the hardware, such as the space and area for the installation of the devices, tables and chairs, device for the identification of patients, computers and blood pressure monitors including the cuffs. The software controls the whole process, including blood pressure measurement and data transmission, storage and analysis. Blood pressure monitors were annually calibrated. The staff were trained for blood pressure measurement and ready for handling any possible problems during measurement.

### Definitions

Blood pressure control was defined as a systolic blood pressure below 140 mmHg and a diastolic blood pressure below 90 mmHg. Regions of the community health centers were classified as urban (non-agricultural registration population percentage greater than 70% and a migrant agricultural population percentage no more than 35%), suburban (non-agricultural registration population percentage greater than 70% and a migrant agricultural population percentage over 35%), and rural (non-agricultural registration population percentage less than 70%). Current smoking was self-reported current tobacco consumption by the patients. Diabetes mellitus was also a self-reported diagnosis. Body mass index was classified as underweight and normal (<24.0 kg/m^2^) and overweight and obesity (≥24.0 kg/m^2^).

Seasons were defined as follows: March to May as spring, June to August as summer, September to November as autumn, and December to February of the following year as winter. Morning blood pressure was defined as that measured from 7AM to 8AM. Noon blood pressure was defined as that measured from 9AM to 4PM. These morning and noon blood pressure measurements were obtained on different days but within the same season. In case of multiple measurements, the two closest ones were selected. Masked uncontrolled morning hypertension was defined as a systolic/diastolic blood pressure uncontrolled in the morning (≥140/90 mmHg) but controlled around noon (<140/90 mmHg).

### Statistical analyses

The SAS software version 9.4 (SAS Institute Inc., Cary, NC, USA) was used for data management and statistical analysis. The R software version 4.2.2 was used for graphics. Continuous variables were described using means and standard deviations, and categorical variables were described using frequencies and percentages. Chi-square test was used to compare discrepancies between groups. Analysis of variance (ANOVA) was used for continuous variables. Unconditional logistic regression analyses were performed for the computation of adjusted control rate of hypertension, the distributions of control rate of hypertension across months and hours, and 95% confidence interval (*95%CI*). The adjusted variables included participants’ characteristics, such as gender, age, region, education level, hypertension course, BMI, and diabetes mellitus. Patients with both morning and non-morning blood pressure readings were selected from each quarter to analyze the prevalence of masked hypertension among those with uncontrolled morning blood pressure. Unconditional stepwise logistic regression was used to analyze the factors influencing masked morning hypertension. A significance level of *P* < 0.05 (two-tailed) indicated statistical significance.

## Results

### Participant characteristics

Participant characteristics are summarized in Table 1. The 343,400 patients with hypertension included 158,870 men (46.3%), and had a mean ( ± SD) age of 70.2 ± 8.1 years, and a mean systolic/diastolic blood pressure of 134.1 ± 18.0/ 75.1 ± 10.4 mmHg. The overall control rate of hypertension was 63.3%, without significant difference between men and women (63.3% vs 63.3%, *P* = 0.661). 14.2% of the participants were current smokers. 28.6% had self-reported diabetes mellitus. The proportion of urban participants was 48.9%. The proportion of participants with elementary or lower educational level 48.5%. The proportion of participants recruited in 2023 was 56.4%.

**Table 1:** Characteristics of the study participants

		All (n = 343,400)	Men (n = 158,870)	Women (n = 184,530)	*P*
Age, years		70.2 ± 8.1	69.8 ± 8.1	70.5 ± 8.0	<0.001
Body mass index, kg/m2		24.2 ± 4.1	24.3 ± 4.2	24.1 ± 4.0	<0.001
Systolic blood pressure, mmHg		134.1 ± 18.0	133.8 ± 17.8	134.3 ± 18.2	<0.001
Diastolic blood pressure, mmHg		75.1 ± 10.4	76.5 ± 10.6	73.8 ± 10.1	<0.001
Heart rate, beats per minute		78.2 ± 11.4	78.4 ± 11.8	78.1 ± 11.1	<0.001
Hypertension control rate, n (%)		217,380 (63.3)	100,630 (63.3)	116,750 (63.3)	0.66
Hypertension duration, years		7.8 ± 5.2	7.7 ± 5.2	8 ± 5.2	<0.001
Current smokers, n (%)		48,905 (14.2)	48,158 (30.3)	747 (0.4)	<0.001
Diabetes mellitus, n(%)		98,121 (28.6)	45,215 (28.5)	52,906 (28.7)	0.17
Region, %	Urban	48.9	46.5	50.9	<0.001
	Suburban	28.2	29.5	27.0	
	Rural	22.9	24.0	22.1	
Education, %	Elementary or lower	48.5	45.8	50.8	<0.001
	Middle school	27.3	27.0	27.5	
	High/specialized secondary	13.4	14.4	12.6	
	Graduate or higher	10.8	12.8	9.1	
Participation year, %	2018	1.4	1.3	1.5	<0.001
	2019	4.3	4.1	4.4	
	2020	8.1	7.9	8.1	
	2021	14.8	14.8	14.7	
	2022	15.1	15.3	14.9	
	2023	56.4	56.6	56.3	

Values are mean ± SD or number (% of the column). *P* values are for the comparison between men and women

### Control rate of hypertension across months of year

**Fig. 1:**
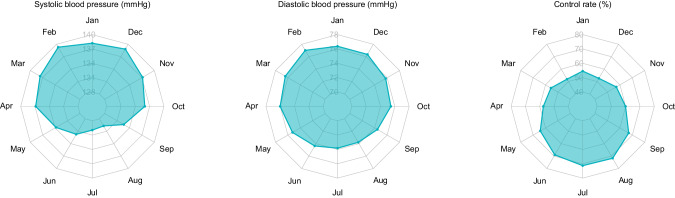
Radar map of mean systolic blood pressure, diastolic blood pressure, and control rate of hypertension across months of year from January to December

The number of participants by month of year and hour of day is given in the Supplementary Table S1. Mean values of systolic/diastolic blood pressure were significantly different across months of year (Fig. 1 and Supplementary Table S2), being highest in February and lowest in August (139.3/77.0 mmHg, 129.7/73.8 mmHg, *P* < 0.001). The differences between the maximum and minimum systolic and diastolic blood pressure were 9.6 mmHg (*95% CI*: 9.3–9.9) and 3.2 mmHg (*95% CI:* 3.0–3.4), respectively. Accordingly, the control rate of hypertension was significantly different across months of year, being lowest in February and highest in August (51.9% vs 71.8%, *P* < 0.001). The difference between the maximum and minimum control rates was 19.9% (*95% CI*: 19.0–20.7).

### Control rates of hypertension across hours of day

**Fig. 2:**
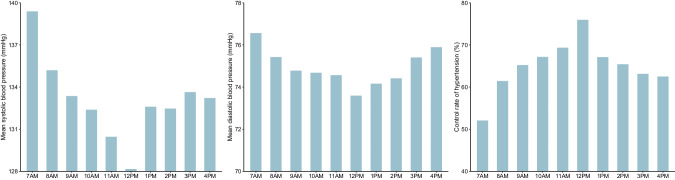
Bar Charts of mean systolic and diastolic blood pressure and the control rate of hypertension across hours of day from 7AM to 4PM

Mean values of systolic/diastolic blood pressure were significantly different across hours of day (Fig. 2 and Supplementary Table S2), being highest at 7 AM and lowest at 12 PM (139.4/76.6 mmHg vs 128.2/73.6 mmHg, *P* < 0.001). The differences between the maximum and minimum systolic and diastolic blood pressure were 11.2 mmHg (*95% CI*: 10.6-11.9) and 3.0 mmHg (*95% CI:* 2.6–3.3), respectively. Accordingly, the control rate of hypertension was also significantly different across hours (*P* < 0.05), being lowest at 7 AM and highest at 12 PM (52.1% vs 76.0%). The difference between the maximum and minimum control rates was 23.9% (*95% CI*: 22.2–25.5).

### Control rates of hypertension across the combination of months of year and hours of day

**Fig. 3:**
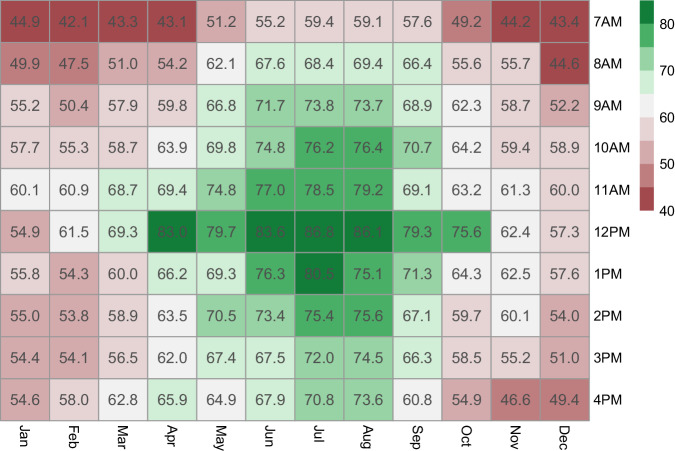
Heat map of the control rate of hypertension across months of year from January to December and hours of day from 7AM to 4PM

When combining month of year and hour of day, mean systolic blood pressure was highest at 7 AM in March and lowest at 12 PM in August (143.6 mmHg vs 121.6 mmHg). The difference between the maximum and minimum systolic blood pressure was 21.9 mmHg (*95% CI*: 20.4–23.4). Mean diastolic blood pressure was highest at 7 AM in April, and lowest at 12 PM in June (77.8 mmHg vs 71.3 mmHg, Supplementary Table S2). The difference between the maximum and minimum diastolic blood pressure was 6.5 mmHg (*95% CI*: 5.3–7.6). The control rate was lowest at 7 AM in February (42.1%, *95% CI*: 37.2-45.1), and highest at 12 PM in July (86.8%, *95% CI*: 82.1–90.5). The difference between the maximum and minimum control rates was 44.7% (*95% CI*: 39.4–50.0, Fig. 3). After adjustment for gender, age, region, education, hypertension duration, BMI, and diabetes mellitus, unconditional logistic regression analyses showed that the control rate of hypertension had a similar pattern as in the unadjusted analysis, displaying a trend of control rate of hypertension being lowest at 7 AM, increasing and peaking at 11 AM to 12 PM, and then gradually decreasing in the afternoon (Supplementary Table S3).

### Prevalence of masked uncontrolled morning hypertension as assessed by noon blood pressure

8516 participants with uncontrolled blood pressure in the early morning had noon blood pressure readings available. 45.7% had controlled blood pressure around noon, and hence masked uncontrolled morning hypertension, with the numbers for spring, summer, autumn, and winter being 47.1%, 50.72%, 43.6%, and 36.3%, respectively (*P* < 0.05). The prevalence of masked uncontrolled morning hypertension in different subgroups is shown in Table 2. Multivariate logistic regression analysis results (Table 3) indicate that women and the summer season were associated with a higher likelihood of masked uncontrolled morning hypertension, whereas ages of 80 and above, diabetes mellitus, and the autumn and winter seasons were associated with a lower likelihood of masked uncontrolled morning hypertension.

**Table 2:** The prevalence of masked morning hypertension between four seasons

		Spring (n = 2978)		Summer (n = 2301)		Autumn (n = 1951)		Winter (n = 1286)	
Variable	Subgroup	Number	Masked morning HT	Number	Masked morning HT	Number	Masked morning HT	Number	Masked morning HT
Gender	Man	1335	43.6†	984	48.6	850	41.1*	635	33.7
	Woman	1643	50.0	1317	52.3	1101	45.6	651	38.9
Age group, years	50-69	1281	49.7*	969	51.7	810	46.9*	623	38.7
	≥70	1697	45.1	1332	50.0	1141	41.3	663	34.1
Region	Urban	903	45.0*	822	46.6*	846	43.6	650	38.3
	Suburban	1214	50.4	852	53.2	737	42.2	471	34.4
	Rural	861	44.7	627	52.8	368	46.5	165	33.9
Education	Elementary or lower	1761	46.8	1321	52.7	1070	42.8	661	36.5
	Middle school	710	50.4	576	48.3	500	43.6	359	34.0
	High/specialized secondary	253	41.1	224	47.8	219	42.0	156	37.8
	Graduate or higher	254	46.1	180	47.8	162	51.2	110	40.9
Hypertension duration, years	<10	1933	47.7	1440	51.6	1349	44.0	925	35.8
	≥10	1045	46.1	861	49.3	602	42.9	361	37.7
Body mass index, kg/m^2^	Underweight and normal ( < 24)	1366	46.4	1077	51.1	964	44.0	655	38.5
	Overweight and obesity ( ≥ 24)	1612	47.7	1224	50.4	987	43.3	631	34.1
Diabetes mellitus	No	1988	48.7*	1580	51.8	1300	44.7	829	37.4
	Yes	990	43.9	721	48.3	651	41.5	457	34.4

Values are number of participants and the prevalence of masked morning hypertension (%). The *P* value is for the comparison between subgroups by season. * *P* < 0.05, †*P* < 0.01

**Table 3:** Multivariate logistic regression analysis of masked morning hypertension between subgroups and seasons

Subgroup		Odds ratio (95%CI)	P
Gender	Man	Ref.	
	Woman	1.24 (1.14–1.35)	<0.001
Age group, years	50-69	Ref.	
	≥80	0.84 (0.77–0.92)	<0.001
Diabetes mellitus	No	Ref.	
	Yes	0.86 (0.79–0.95)	0.002
Season	Spring	Ref.	
	Summer	1.15 (1.03–1.28)	0.012
	Autumn	0.87 (0.77–0.97)	0.016
	Winter	0.64 (0.56–0.73)	<0.001

Masked morning hypertension was defined as office morning blood pressure ≥140/90 mmHg and office non-morning blood pressure in daytime <140/90 mmHg. Multivariate logistic regression analysis was used to assess masked morning hypertension in subgroups (gender, age group, region, education, hypertension duration, body mass index and diabetes mellitus) and seasons. The stepwise method was used for the variable selection, and finally included in the model were gender, age group, diabetes mellitus and season

*CI* confidence interval.

## Discussion

Our real-world study in a large number of patients with hypertension showed that the control rate of hypertension varied across months of year from January to December and hours of day from 7 AM to 4 PM. Systolic and diastolic blood pressures were highest in February and at 7 AM, and lowest in August and at noon. An immediate implication is that the evaluation of blood pressure control might have to take months of year and hours of day into account.

Our finding is generally consistent with the results of several previous studies on seasonal variation in blood pressure. In about 500,000 participants enrolled in the Kadoorie Biobank cohort study in China, blood pressure tended to be higher in winter than in other seasons, especially summer [18]. Similarly, in a large, national, electronic health records-based study from the US [19], the control rate of hypertension was higher in warmer than colder seasons, being 32.0% in the months from April to June and 62.3% in the months from July to August. The mechanisms, though complex, might be related to ambient temperature in four-season countries, such as China, being much higher in temperature in summer than in winter and other seasons (Supplementary Table S4) [20].

Our finding on the hourly variation within a day is also in line with the results of previous studies. Several previous studies in Asia showed that blood pressure in the morning was significantly higher than in the evening [21]. Few studies compared morning with noon blood pressure. Because of the well-known phenomenon of postprandial hypotension [22], blood pressure at noon, especially after lunch, must be lower than that was measured at the rest hours of the day. The results of the present study showed that nearly half of the participants with uncontrolled blood pressure in the morning had masked hypertension, especially higher in spring and summer. It was also more likely to occur in women, those aged 50-69, and non-diabetic patients. If blood pressure were not measured in the morning and around noon as well for these participants, there would be a large number of missed diagnoses. It is apparently preferrable to perform home blood pressure monitoring for the assessment of morning blood pressure. Nonetheless, without home blood pressure measurement, it is reasonable to make medical appointment in the morning, whenever possible, for morning blood pressure control in hypertensive patients.

To the best of our knowledge, this is the first study that investigated the combined effect of months of year and hours of day. A key finding is that there is indeed a combined effect of months of year and hours of day. Blood pressure was lower at noon, especially in the months in summer. In contrast, blood pressure was higher in the morning than the rest hours of the day, especially in the months in winter. The results suggest that the control rate of hypertension might have to take into account seasons and hours of the day.

### Strengths and limitations

Our study should be interpreted within the context of its strengths and limitations. The sample size of our study was large. More importantly, blood pressure measurement was performed on an automated office blood pressure platform with the possibility of automatic transmission of the blood pressure readings to a digital platform. Nonetheless, our study participants were not randomly selected, and hence may not be perfectly representative of the hypertensive population in Shanghai. In addition, our study was cross-sectional, and only included blood pressure on a single occasion.

### Perspective of Asia

Asians, compared with Westerners, had a greater morning blood pressure surge [23] and higher prevalence of nocturnal hypertension [24] and had different patterns in the difference between clinic and home blood pressures during different time of day [25]. Our finding on the hourly and monthly variation in the evaluation of blood pressure control in the Chinese population provides further support for the clinical relevance of morning blood pressure in the management of hypertension in Asians [26], especially in cold seasons.

## Conclusions

The control rate of hypertension varies across months of year and hours of day. If confirmed in future studies, especially with regard to hard clinical outcomes, our study results suggest that the evaluation of blood pressure control has to take into season and hour of day into account. With the increasing use of digital platforms in the management of hypertension, these seasonal and hourly factors must and can be considered.

## Supplementary information

Supplementary Table S1

Supplementary Table S2

Supplementary Table S3

Supplementary Table S4
