# Supplementary material for: The control rate of hypertension across months of year and hours of day in a large real-world database
Source: Hypertens Res. 2024 Aug 21;47(11):2981–8. doi: 10.1038/s41440-024-01817-1 (PMC11534683; doi:10.1038/s41440-024-01817-1)
Supplement: Supplementary file 1 — Supplementary Table S1 [file 41440_2024_1817_MOESM1_ESM.docx]

Supplementary Table S1. Number of patients by months of year and hours of day

| Month | Jan | Feb | Mar | Apr | May | Jun | Jul | Aug | Sep | Oct | Nov | Dec | All |
| --- | --- | --- | --- | --- | --- | --- | --- | --- | --- | --- | --- | --- | --- |
| 7AM | 558 | 776 | 3,091 | 5,011 | 4,906 | 5,029 | 4,832 | 5,531 | 4,493 | 1,446 | 1,780 | 988 | 38,441 |
| 8AM | 1,927 | 3,283 | 6,060 | 7,465 | 7,892 | 9,058 | 10,187 | 12,998 | 10,516 | 4,143 | 4,195 | 3,150 | 80,874 |
| 9AM | 2,590 | 3,807 | 6,165 | 6,370 | 6,571 | 7,480 | 8,357 | 10,763 | 9,784 | 4,640 | 4,713 | 3,871 | 75,111 |
| 10AM | 2,399 | 2,893 | 4,238 | 3,829 | 3,894 | 4,609 | 4,861 | 6,655 | 6,487 | 3,570 | 3,675 | 3,491 | 50,601 |
| 11AM | 632 | 560 | 878 | 878 | 743 | 913 | 871 | 1,264 | 1,075 | 798 | 764 | 976 | 10,352 |
| 12PM | 113 | 156 | 319 | 258 | 246 | 299 | 266 | 438 | 208 | 221 | 210 | 178 | 2,912 |
| 1PM | 1,094 | 1,497 | 2,149 | 1,734 | 1,785 | 1,975 | 1,820 | 2,188 | 2,478 | 1,589 | 1,720 | 1,371 | 21,400 |
| 2PM | 1,678 | 1,879 | 2,672 | 2,366 | 2,531 | 2,882 | 3,229 | 4,126 | 3,810 | 2,455 | 2,627 | 2,314 | 32,569 |
| 3PM | 1,295 | 1,190 | 1,816 | 1,680 | 1,667 | 2,058 | 2,519 | 3,395 | 2,779 | 1,949 | 2,031 | 1,995 | 24,374 |
| 4PM | 467 | 331 | 524 | 472 | 436 | 555 | 730 | 1,115 | 663 | 468 | 446 | 559 | 6,766 |
| All | 12,753 | 16,372 | 27,912 | 30,063 | 30,671 | 34,858 | 37,672 | 48,473 | 42,293 | 21,279 | 22,161 | 18,893 | 343,400 |

Values are number of patients with blood pressure measurement.
