# Supplementary material for: The control rate of hypertension across months of year and hours of day in a large real-world database
Source: Hypertens Res. 2024 Aug 21;47(11):2981–8. doi: 10.1038/s41440-024-01817-1 (PMC11534683; doi:10.1038/s41440-024-01817-1)
Supplement: Supplementary file 2 — Supplementary Table S2 [file 41440_2024_1817_MOESM2_ESM.docx]

Supplementary Table S2. Mean systolic and diastolic blood pressure in mmHg across months of year and hours of day

| Month |  | Jan | Feb | Mar | Apr | May | Jun | Jul | Aug | Sep | Oct | Nov | Dec | All |
| --- | --- | --- | --- | --- | --- | --- | --- | --- | --- | --- | --- | --- | --- | --- |
| 7AM | Systolic | 141.9±16.9 | 142.5±15.8 | 143.6±17.6 | 143.3±17.7 | 139.7±17 | 138.1±17.5 | 136.2±18.1 | 136.5±18.3 | 137.1±17.3 | 141.1±17.6 | 142.0±17.2 | 142.4±16.5 | 139.4±17.8 |
|  | Diastolic | 76.7±10.3 | 76.7±9.8 | 77.6±10.4 | 77.8±10.2 | 77.0±10.0 | 76.5±10.1 | 75.7±10.5 | 75.8±10.7 | 75.5±10.2 | 76.4±10.4 | 77.1±10.6 | 77.6±10.7 | 76.6±10.4 |
| 8AM | Systolic | 140±16.8 | 141.5±17.3 | 139.7±17.5 | 138.7±17.5 | 135.2±17.1 | 132.8±17.1 | 131.8±17.2 | 131.4±17.5 | 133.2±17.2 | 137.7±17.2 | 138.1±17.2 | 141.8±17.7 | 135.2±17.6 |
|  | Diastolic | 77.1±10.5 | 77.9±10.3 | 77.4±10.1 | 76.8±10.2 | 75.7±10.2 | 74.8±9.9 | 74.4±10.4 | 73.8±10.4 | 74.7±10.2 | 75.6±10.3 | 75.9±10.5 | 77.3±10.8 | 75.4±10.3 |
| 9AM | Systolic | 137.9±16.5 | 139.5±17.1 | 136.8±17.3 | 135.7±17.4 | 132.6±16.9 | 130.8±16.8 | 129.2±17.4 | 129.4±17.3 | 131.8±17.1 | 135.3±17.1 | 136.5±17.3 | 138.9±17.5 | 133.4±17.5 |
|  | Diastolic | 75.9±10.7 | 77.2±10.2 | 76.2±10.5 | 75.6±10.2 | 75.0±10.0 | 74.0±10.0 | 73.3±10.1 | 73.4±10.2 | 74.1±10.4 | 75.1±10.3 | 75.5±10.6 | 76.4±10.5 | 74.8±10.3 |
| 10AM | Systolic | 136.9±17.4 | 138±17.7 | 135.8±17.3 | 134.4±17.5 | 131.5±16.9 | 129.7±16.5 | 127.6±17.7 | 127.5±17.3 | 130.7±17.0 | 134±16.8 | 136.1±17.6 | 136.4±17.6 | 132.4±17.6 |
|  | Diastolic | 76.0±10.7 | 76.7±10.4 | 76.0±10.5 | 75.2±10.3 | 74.8±10.3 | 73.6±9.9 | 72.8±10.1 | 73.4±10.2 | 74.1±10.4 | 75.3±10.2 | 75.8±10.4 | 75.5±10.5 | 74.7±10.4 |
| 11AM | Systolic | 135.8±17.2 | 134.2±17.1 | 131.6±18.0 | 129.7±19.4 | 128.5±17.8 | 126.8±17.3 | 124.7±17.9 | 124.7±17.5 | 130.8±17.6 | 133.8±17.9 | 134.6±17.3 | 135.8±17.1 | 130.5±18.1 |
|  | Diastolic | 74.6±11.2 | 75.4±10.7 | 75.3±9.9 | 74.6±10.2 | 74.4±9.9 | 73.1±10.1 | 73.2±10.5 | 74.1±10.3 | 74.4±10.3 | 76.5±10.8 | 75±10.5 | 75.0±10.4 | 74.6±10.4 |
| 12PM | Systolic | 138.0±18.4 | 137.4±19.6 | 130.7±19.1 | 125.3±17.8 | 126.2±17.5 | 123.6±16.4 | 124.3±14.1 | 121.6±14.6 | 127.3±15.3 | 130.4±15.7 | 133.3±17.7 | 138.1±17.3 | 128.2±17.6 |
|  | Diastolic | 75.6±10.6 | 75.3±10.8 | 73.6±10.5 | 72.4±9.3 | 72.4±9.4 | 71.3±9.5 | 73.0±8.7 | 74.9±9.9 | 72.7±10.7 | 74.2±9.9 | 72.9±9.9 | 76.8±10.1 | 73.6±10.0 |
| 1PM | Systolic | 137.9±16.6 | 139.1±17.1 | 136.0±16.9 | 133.3±18.1 | 131.5±17.4 | 128.3±16.6 | 125.4±17.2 | 127.7±17.5 | 131.1±17 | 134.8±16.5 | 135.3±17.6 | 137.2±17.1 | 132.6±17.6 |
|  | Diastolic | 76.3±10.3 | 76.4±10.5 | 75.3±10.3 | 74.3±10.6 | 73.8±9.9 | 72.8±9.4 | 72.3±9.4 | 73.0±10.5 | 73.3±10.2 | 74.4±10.1 | 74.5±10.9 | 75.5±10.9 | 74.2±10.3 |
| 2PM | Systolic | 137.9±17.1 | 138.4±17.6 | 136.2±18.0 | 133.9±18.5 | 130±18.1 | 128.1±18.1 | 126.0±18.8 | 126.6±18.8 | 132±18.0 | 135.7±17.5 | 136.5±17.7 | 138.4±17.1 | 132.5±18.6 |
|  | Diastolic | 75.9±10.4 | 76.3±10.6 | 75.6±10.6 | 74.8±10.2 | 74.0±9.9 | 73.0±10.3 | 72.9±10.2 | 72.8±10.4 | 73.9±10.2 | 75.3±10.5 | 75.3±10.5 | 76.2±10.3 | 74.4±10.4 |
| 3PM | Systolic | 138.7±18 | 138.9±17.5 | 137.0±18.5 | 134.2±18.3 | 131.8±18.3 | 131.4±18.2 | 128.6±18.4 | 126.8±18.6 | 132.9±17.8 | 136.5±18.4 | 137.6±17.7 | 139.6±17.9 | 133.6±18.7 |
|  | Diastolic | 77.4±10.5 | 77.6±10.5 | 76.4±10.8 | 75.4±10.5 | 74.6±10.3 | 74.5±10.3 | 74.3±10.4 | 73.5±10.5 | 74.8±10.6 | 76.2±10.6 | 76.7±10.4 | 76.9±10.6 | 75.4±10.6 |
| 4PM | Systolic | 138.9±17.6 | 136.6±18.9 | 133.6±20.0 | 131.7±19.0 | 131.4±20.1 | 131.5±18.6 | 128.3±19.1 | 126.5±19.1 | 134.8±19.1 | 137.1±19.1 | 140.3±18.7 | 139.5±17.9 | 133.2±19.5 |
|  | Diastolic | 79.1±10.8 | 77.0±10.3 | 75.8±10.2 | 75.8±10.2 | 74.9±10.4 | 74.1±10.4 | 73.8±10.2 | 75.2±11.1 | 75.8±10.7 | 76.0±11.1 | 78.2±11.2 | 77.6±10.8 | 75.9±10.7 |
| All | Systolic | 138.2±17.1 | 139.3±17.4 | 137.6±17.8 | 136.9±18.2 | 133.8±17.6 | 131.7±17.4 | 130±18.0 | 129.7±18 | 132.6±17.4 | 136.0±17.4 | 137.2±17.6 | 138.8±17.5 | 134.1±18.0 |
|  | Diastolic | 76.4±10.6 | 77.0±10.4 | 76.4±10.4 | 76.0±10.3 | 75.3±10.1 | 74.4±10.0 | 73.8±10.3 | 73.8±10.4 | 74.4±10.3 | 75.5±10.4 | 75.8±10.6 | 76.4±10.6 | 75.1±10.4 |
