# Supplementary material for: The control rate of hypertension across months of year and hours of day in a large real-world database
Source: Hypertens Res. 2024 Aug 21;47(11):2981–8. doi: 10.1038/s41440-024-01817-1 (PMC11534683; doi:10.1038/s41440-024-01817-1)
Supplement: Supplementary file 3 — Supplementary Table S3 [file 41440_2024_1817_MOESM3_ESM.docx]

Supplementary Table S3. Adjusted control rate of hypertension in percentages of patients with 95% confidence intervals across months of year and hours of day

| Month | Jan | Feb | Mar | Apr | May | Jun | Jul | Aug | Sep | Oct | Nov | Dec |
| --- | --- | --- | --- | --- | --- | --- | --- | --- | --- | --- | --- | --- |
| 7AM | 44.1 (39.4-48.9) | 41.1 (37.2-45.1) | 43.1 (40.9-45.5) | 44.8 (42.8-46.9) | 52.2 (50-54.3) | 56.1 (54-58.2) | 60.4 (58.4-62.4) | 59.9 (58.1-61.7) | 56.2 (54.3-58.2) | 47 (43.8-50.2) | 41.8 (39-44.7) | 42.5 (38.9-46.2) |
| 8AM | 48.4 (45.2-51.6) | 46 (43.4-48.7) | 49.9 (47.9-51.9) | 55.7 (53.8-57.6) | 62.4 (60.6-64.1) | 68 (66.3-69.6) | 69 (67.5-70.6) | 69.7 (68.3-71) | 65 (63.5-66.5) | 53.9 (51.5-56.4) | 52.8 (50.5-55) | 43.2 (40.5-45.8) |
| 9AM | 53.6 (50.6-56.6) | 48.4 (45.8-51) | 56.4 (54.4-58.3) | 60.3 (58.4-62.1) | 66.3 (64.5-68) | 71.7 (70.1-73.3) | 74.1 (72.6-75.5) | 73.4 (72.1-74.7) | 67.5 (66-69) | 60.5 (58.2-62.8) | 55.8 (53.6-58) | 50.1 (47.5-52.7) |
| 10AM | 55.7 (52.6-58.7) | 52.8 (50.1-55.6) | 56.8 (54.7-58.9) | 63.4 (61.3-65.4) | 68.7 (66.7-70.6) | 74.4 (72.6-76.1) | 76.4 (74.8-78) | 75.8 (74.4-77.2) | 69.3 (67.7-70.9) | 62.4 (60-64.8) | 56.3 (54-58.7) | 56.5 (53.8-59.1) |
| 11AM | 57.8 (53.1-62.4) | 57.6 (52.9-62.2) | 67.1 (63.6-70.5) | 68.6 (65.1-71.9) | 73 (69.3-76.3) | 76.5 (73.4-79.4) | 79.1 (76.1-81.8) | 78.6 (76.1-81) | 68.1 (64.9-71) | 62.2 (58.3-66) | 58.4 (54.3-62.3) | 57.5 (53.7-61.3) |
| 12PM | 52.6 (43-62) | 60.6 (52.4-68.2) | 68.4 (62.9-73.5) | 83.8 (78.8-87.8) | 79.6 (74-84.3) | 83.6 (78.8-87.4) | 86.8 (82.1-90.5) | 86.1 (82.5-89.1) | 78.2 (71.8-83.4) | 72.9 (66.1-78.7) | 57.6 (50.4-64.5) | 54.5 (46.8-62.1) |
| 1PM | 53.6 (49.8-57.4) | 52.3 (49-55.6) | 58.1 (55.5-60.7) | 66.1 (63.4-68.6) | 69.1 (66.5-71.5) | 75.9 (73.6-78) | 80.4 (78.3-82.4) | 74.7 (72.6-76.7) | 69.2 (67-71.3) | 61.6 (58.5-64.6) | 58.7 (55.8-61.5) | 55.2 (51.8-58.5) |
| 2PM | 52.6 (49.2-56) | 51.5 (48.4-54.6) | 57 (54.6-59.4) | 62.5 (60.1-64.9) | 69.3 (67-71.5) | 72.9 (70.9-74.9) | 75.3 (73.4-77) | 75 (73.4-76.6) | 65.3 (63.3-67.2) | 57.8 (55-60.5) | 57 (54.4-59.5) | 51.2 (48.2-54.2) |
| 3PM | 51.7 (48-55.4) | 51.1 (47.5-54.6) | 54.1 (51.3-56.9) | 60.5 (57.7-63.3) | 66 (63.2-68.6) | 66.6 (64.1-69) | 71.8 (69.7-73.9) | 73.6 (71.8-75.4) | 64.4 (62.2-66.6) | 57 (54.1-59.9) | 51.8 (48.9-54.6) | 47.9 (44.8-51) |
| 4PM | 52.3 (47.1-57.5) | 54.9 (49-60.6) | 60.8 (56.2-65.2) | 64.4 (59.7-68.9) | 62.3 (57.3-67.1) | 67.3 (62.9-71.3) | 71.2 (67.6-74.6) | 73 (70.1-75.7) | 59.8 (55.8-63.7) | 54.4 (49.4-59.3) | 43.9 (39-48.9) | 46.5 (41.8-51.2) |
