# Supplementary material for: The control rate of hypertension across months of year and hours of day in a large real-world database
Source: Hypertens Res. 2024 Aug 21;47(11):2981–8. doi: 10.1038/s41440-024-01817-1 (PMC11534683; doi:10.1038/s41440-024-01817-1)
Supplement: Supplementary file 4 — Supplementary Table S4 [file 41440_2024_1817_MOESM4_ESM.docx]

Supplementary Table S4. Mean [atmospheric temperature](javascript: void(0)) and relative humidity across months of year and hours of day

|  | Month | Jan | Feb | Mar | Apr | May | Jun | Jul | Aug | Sep | Oct | Nov | Dec |
| --- | --- | --- | --- | --- | --- | --- | --- | --- | --- | --- | --- | --- | --- |
| Temperature, ℃ | 7AM | 3.5 | 4.5 | 9.2 | 13.8 | 19.3 | 23.3 | 26.8 | 27.3 | 23.2 | 17.3 | 12.6 | 5.2 |
|  | 8AM | 4.0 | 5.4 | 10.5 | 15.2 | 20.4 | 24.2 | 27.6 | 28.1 | 24.3 | 18.7 | 13.7 | 5.9 |
|  | 9AM | 5.5 | 6.8 | 11.7 | 16.4 | 21.4 | 25.0 | 28.3 | 28.9 | 25.1 | 19.9 | 15.1 | 7.6 |
|  | 10AM | 6.7 | 7.9 | 12.8 | 17.5 | 22.3 | 25.7 | 28.9 | 29.6 | 25.9 | 20.8 | 16.1 | 8.8 |
|  | 11AM | 7.5 | 8.7 | 13.7 | 18.3 | 23.0 | 26.3 | 29.5 | 30.1 | 26.4 | 21.4 | 16.8 | 9.7 |
|  | 12PM | 8.1 | 9.3 | 14.4 | 18.8 | 23.5 | 26.7 | 29.9 | 30.5 | 26.7 | 21.8 | 17.3 | 10.2 |
|  | 1PM | 8.5 | 9.7 | 14.7 | 19.1 | 23.8 | 26.9 | 30.1 | 30.8 | 26.9 | 22.0 | 17.5 | 10.5 |
|  | 2PM | 8.5 | 9.8 | 14.8 | 19.2 | 23.8 | 27.0 | 30.1 | 30.8 | 26.8 | 22.0 | 17.5 | 10.5 |
|  | 3PM | 8.3 | 9.6 | 14.7 | 19.0 | 23.7 | 26.9 | 30.1 | 30.8 | 26.7 | 21.7 | 17.2 | 10.2 |
|  | 4PM | 7.9 | 9.2 | 14.2 | 18.5 | 23.2 | 26.5 | 29.7 | 30.4 | 26.3 | 21.2 | 16.7 | 9.7 |
| Relative humidity, % | 7AM | 83.6 | 85.5 | 87.2 | 84.1 | 83.7 | 87.9 | 88.5 | 87.4 | 87.9 | 82.6 | 84.5 | 81.7 |
|  | 8AM | 82.0 | 82.4 | 81.6 | 77.0 | 77.9 | 83.6 | 84.5 | 82.6 | 82.4 | 76.7 | 80.6 | 79.7 |
|  | 9AM | 75.7 | 75.7 | 76.2 | 71.6 | 73.1 | 79.7 | 81.2 | 78.6 | 77.6 | 70.3 | 73.6 | 72.7 |
|  | 10AM | 69.7 | 70.3 | 71.3 | 67.1 | 69.2 | 76.5 | 78.3 | 75.2 | 73.7 | 65.2 | 67.8 | 66.5 |
|  | 11AM | 65.3 | 66.3 | 67.4 | 63.9 | 66.1 | 74.0 | 76.1 | 72.8 | 71.1 | 61.9 | 63.9 | 62.1 |
|  | 12PM | 62.5 | 63.9 | 64.7 | 61.7 | 64.1 | 72.3 | 74.7 | 71.0 | 69.4 | 60.0 | 61.5 | 59.2 |
|  | 1PM | 61.0 | 62.4 | 63.1 | 60.6 | 63.1 | 71.3 | 74.0 | 70.0 | 68.5 | 58.9 | 60.2 | 57.6 |
|  | 2PM | 60.6 | 61.8 | 62.5 | 60.2 | 63.1 | 71.1 | 74.0 | 69.7 | 68.7 | 58.7 | 60.0 | 57.1 |
|  | 3PM | 61.5 | 62.1 | 62.5 | 60.7 | 63.4 | 71.3 | 73.8 | 69.3 | 68.6 | 59.2 | 61.2 | 58.1 |
|  | 4PM | 63.3 | 63.8 | 64.5 | 62.8 | 65.5 | 73.3 | 75.8 | 71.4 | 70.5 | 62.1 | 64.3 | 60.5 |
